# CT-based habitat radiomics for predicting treatment response to neoadjuvant chemoimmunotherapy in esophageal cancer patients

**DOI:** 10.3389/fonc.2024.1418252

**Published:** 2024-12-03

**Authors:** Weibo Kong, Junrui Xu, Yunlong Huang, Kun Zhu, Long Yao, Kaiming Wu, Hanlin Wang, Yuhang Ma, Qi Zhang, Renquan Zhang

**Affiliations:** ^1^ Department of Thoracic Surgery, First Affiliated Hospital of Anhui Medical University, Hefei, China; ^2^ Department of Gastrointestinal Surgery, The First Affiliated Hospital of Anhui Medical University, Hefei, China; ^3^ Department of Radiology, The First Affiliated Hospital of Anhui Medical University, Hefei, China

**Keywords:** esophageal cancer, habitat, radiomics, neoadjuvant chemoimmunotherapy, pathologic complete response

## Abstract

**Introduction:**

We used habitat radiomics as an innovative tumor biomarker to predict the outcome of neoadjuvant therapy for esophageal cancer.

**Methods:**

This was a two-center retrospective clinical study in which pretreatment CT scans of 112 patients with esophageal cancer treated with neoadjuvant chemoimmunotherapy and surgery between November 2020 and July 2023 were retrospectively collected from two institutions. For training (n = 85) and external testing (n = 27), patients from both institutions were allocated. We employed unsupervised methods to delineate distinct heterogeneous regions within the tumor area.

**Results:**

To represent the prediction effect of different models, we plotted the AUC curves. The AUCs of the habitat models were 0.909 (0.8418–0.9758, 95% CI) and 0.829 (0.6423–1.0000, 95% CI) in the training and external test cohorts, respectively. The AUCs of the nomogram models were 0.914 (0.8483–0.9801, 95% CI) and 0.849 (0.6752–1.0000, 95% CI) in the training and external test cohorts, respectively.

**Discussion:**

The results revealed that the model based on habitat data outperforms traditional radiomic analysis models. In addition, when the model is combined with clinical features, it improves the predictive accuracy of pathological complete response in patients undergoing neoadjuvant chemoimmunotherapy.

## Introduction

Esophageal cancer (EC) is the seventh most common cancer and the sixth leading cause of cancer death worldwide (1). Approximately 544,000 deaths from esophageal cancer were reported in 2020. The 5-year survival rate for all stages of esophageal cancer combined is approximately 20% (2). China has a high incidence of esophageal cancer, of which squamous cell carcinoma (SCC) is the main tissue subtype (3).

The clinical outcomes of patients have dramatically changed with the application of anti-programmed death-ligand 1 (PD-L1) antibodies in the treatment of EC. Recently, an increasing number of studies have reported the use of chemoimmunotherapy in the neoadjuvant treatment of locally advanced EC. Wang et al. retrospectively analyzed the esophagectomy of patients with esophageal squamous cell carcinoma after neoadjuvant chemotherapy (NCT) or neoadjuvant chemoimmunotherapy(NACI). Compared with NCT alone in the treatment of locally advanced esophageal squamous cell carcinoma, NACI has a superior pathological response rate and disease-free survival (DFS) and overall survival (OS) of 3 years (4). For patients with locally advanced EC, neoadjuvant chemoimmunotherapy is widely used as a first-line treatment (5, 6). Nevertheless, the response of esophageal cancer patients to NACI is highly variable, with an average of 20% to 40% of patients estimated to have pathologic complete response(PCR) (5). Previous studies have shown that biomarkers such as CA-125, VEGF, CA-199, and Ki-67 are predictive of esophageal cancer (7, 8). An alternative approach is medical imaging, which is essential in the diagnosis of tumors prior to neoadjuvant therapy and in the analysis of tumor heterogeneity (9, 10).

The heterogeneity of esophageal cancer is a challenge for clinical treatment decisions and aftercare support. Such heterogeneity could also explain the variable response to NACI in esophageal cancer patients (11). Radiomics is a process for transforming images into high-volume extractable data. Compared with several of the commonly used quantitative parameters (e.g., CT values) in patient CT scans, current advances in radiomics have demonstrated its potential added value in tumor identification and prognostic assessment (12).

Recent studies have shown that the radiomic model can effectively predict the pathological response of advanced tumors after neoadjuvant therapy. Liu et al. established and verified a machine learning model based on MR radiology, which can accurately predict the PCR of patients with esophageal squamous cell carcinoma after neoadjuvant chemotherapy and radiotherapy(nCRT) (13). Qi et al. used a machine learning model from 18F-FDG PET and enhanced CT to predict the PCR of esophageal squamous cell carcinoma after nCRT and treatment with an anti-PD-1 inhibitor (14). Ou et al. established a CT radiomic model to predict the efficacy of paclitaxel combined with cisplatin in the treatment of advanced esophageal squamous cell carcinoma (15).

Most studies related to radiomics, however, usually treat the whole tumor as the object of study, with little consideration given to extracting features from different subregions with different metabolic profile. Caii et al. combines deep learning and habitat radiomics techniques to establish a non-invasive model for predicting immunotherapy response in patients with advanced non-small cell lung cancer (16). Therefore, we made a hypothesis that habitat analysis can predict the effect of neoadjuvant therapy for esophageal cancer. In this study, we divided tumors into subregions containing clusters of voxels with similar features. This approach better quantifies the heterogeneity within the tumor. Habitat radiomics is based on the presumption that identified subregions composed of voxels with similar imaging features will share common tumor characteristics (17–20). Therefore, habitat analysis may be a valuable biomarker for predicting clinical outcomes (called habitats).

**Figure 1:**
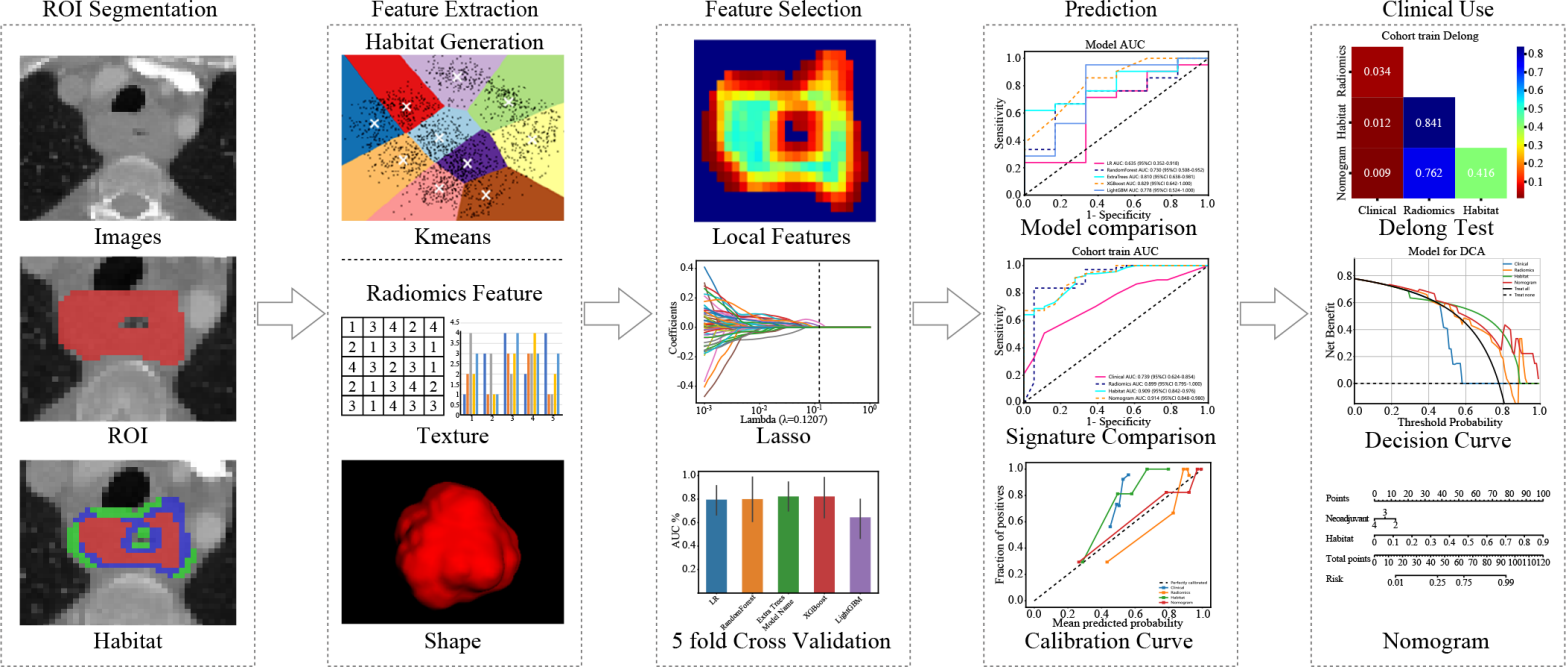
Overall workflow of this study.

In summary, our study introduces a new habitat model that combines robust feature selection with clinical usability. These advances significantly improve the prediction of neoadjuvant therapy outcomes. (**Figure 1**)

## Materials and methods

### Datasets

This retrospective study was approved by the institutional review board. Two cohorts of EC patients from The First Affiliated Hospital of Anhui Medical University and The First Affiliated Hospital of Anhui Medical University Gaoxin Branch were retrospectively identified. Written informed consent was waived because of the retrospective nature of this study.

**Figure 2:**
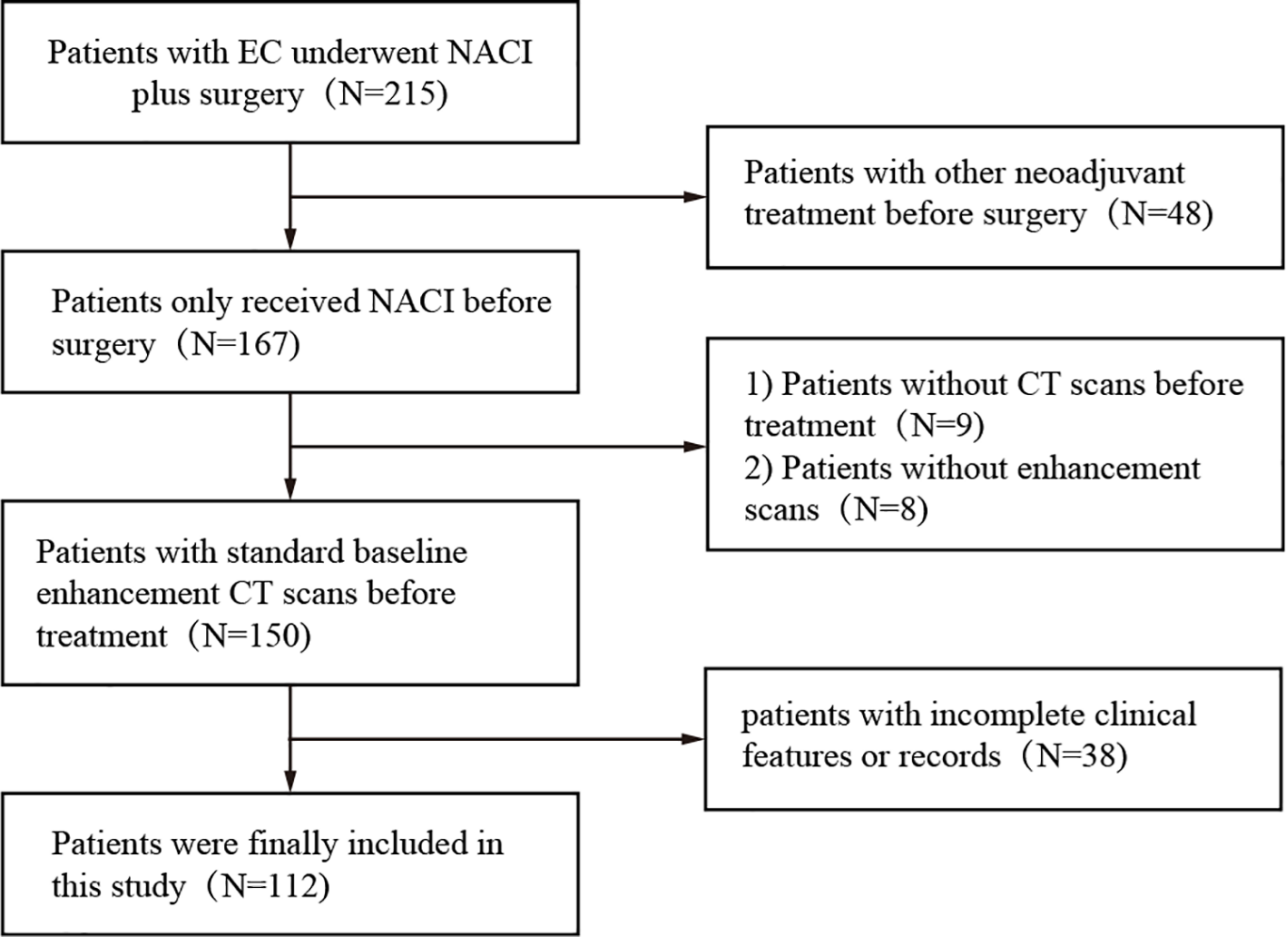
Flow chart of patient selection process.

The dataset used in this study consisted of pretreatment CT scans of patients who received neoadjuvant chemoimmunotherapy between January 2019 and May 2023 at two centers, both of which are academic medical centers. The inclusion criteria for all patients were as follows: a) biopsy-proven resectable esophageal cancer (clinical stage cT1-4N1-3M0 or cT3-4N0M0), b) enhanced CT within one month prior to neoadjuvant therapy, and c) postoperative pathologic assessment of the response to neoadjuvant therapy. The exclusion criteria were (a) missing or poor-quality CT, (b) previous history of cancer, (c) incomplete NACI, (d) lack of complete pre-NACI histopathological data, (e) failure to undergo NACI, and (f) lack of information on pathologic response. The patients received a NACI regimen involving camrelizumab (200 mg) in combination with albumin paclitaxel (260 mg/m²) and cisplatin (60 mg/m² Q3W) (**Figure 2**).

### Image acquisition

All participants were informed and signed the consent form for contrast agent injection before receiving an enhanced CT scan. CT scans were obtained using a 256-slice CT scanner (GE Healthcare, Revolution CT, USA). Preparation for the examination consisted of fasting for more than 8 hours before the examination and breath-holding exercises. The scanning parameters were as follows: tube voltage, 120 kV; tube current, automatic milliampere second technology; pitch, 1.375/1.1; field of view (FOV), 500 mm; 512 mm × 512 mm matrix; scan thickness, 0.625 mm— to 5 mm; and scan spacing, 0.625 mm— to 5 mm. The scan area included at least the pharynx to the upper edge of the pelvis. For enhanced scanning, 90–100 mL of nonionic contrast agent was injected through the elbow vein via a high-pressure syringe (GE Medical Systems, iohexol, 300 mg/mL).

### Image processing and segmentation

Artery phase and vein phase CT images were isometrically resampled using tri-linear interpolation in ITK-SNAP (version 3.8.0) software with a voxel size of 1 mm×1 mm×1 mm. On the CT image, the region of interest (ROI) is outlined in layers, and the 3D ROIs are finally combined to form a volume of interest (VOI). During segmentation, the esophageal lumen and mucosa, periesophageal fat and blood vessels should be avoided. The segmentation of VOIs was performed by a radiologist with 5 years of experience in diagnostic chest imaging. For interobserver consistency analysis, when the radiologist performed the first segmentation of the full dataset, another radiologist (with 10 years of experience in diagnostic chest imaging) performed the segmentation of the selected 30 patients at the same time. For intraclass correlation coefficient (ICC) analysis, the segmentation was repeated 1 month after the initial segmentation.

### Data preprocessing

Our study implemented crucial techniques to enhance medical image analysis. We standardized the pixel values to a range of 0— to 2048, reducing the impact of outliers. Additionally, we employed fixed-resolution resampling to achieve a uniform voxel spacing of , ensuring precise comparisons across images. These refinements significantly bolstered the reliability and accuracy of our analysis.

### Histopathologic response evaluation

Two trained pathologists (with 10 and 20 years of experience) evaluated the resected surgical specimens and scored the response according to Mandal’s tumor regression grade (TRG) five-grade scale. If the residual tumor is graded TRG 1 (i.e., no remaining cancer cells are replaced by extensive fibrosis), this indicates PCR (21).

### Habitat-oriented radiomics approach

**Figure 3:**
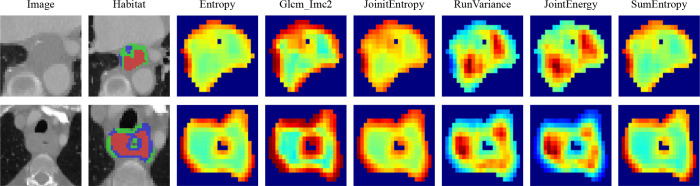
Presents the peritumoral regions generated and the habitat regions generated.

Local features, including measures such as local entropy and energy values, were computed for every voxel inside the VOI, forming feature vectors that encapsulated different voxel properties. These features were calculated using a 3 × 3 × 3 nonoverlapping moving window applied to each voxel in the CT images. This process generated a 19-dimensional feature vector for every voxel. Subsequently, employing the K-means method, we partitioned the VOI into three distinct regions for each sample. To prevent overparameterization and draw on insights from prior habitat-related literature, we preset three clustering centers as the number of habitat regions (22). We posit that experimenting with different numbers of clustering centers may enhance model performance (**Figure 3**).

**Figure 4:**
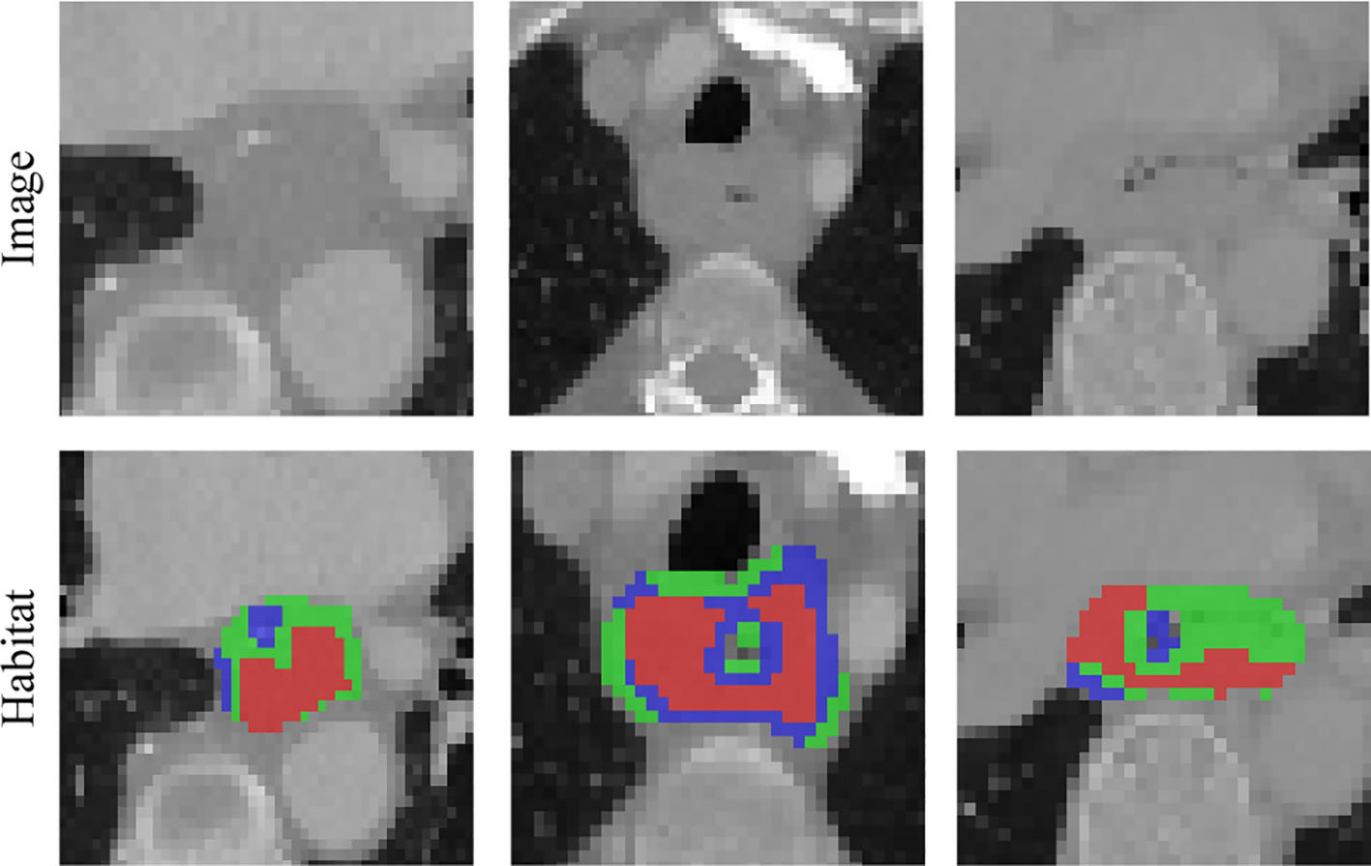
Various samples with three clustering labels are shown, but with different labels assigned. Our clustering algorithm categorizes regions into interior, intermediate, and edge regions. In the figure, the red area to the left represents the intermediate region while the green area to the right represents the middle region.

The manual features in this study include three categories: geometric features, intensity features, and texture features. For the internal analysis of the tumor (the VOI was considered as a whole), we extracted the radiomic features described above. For the habitat features, we extracted features for each subregion. Since the clustering algorithm used is unsupervised, there is no guarantee that each subregion retains the same label after clustering. For example, as shown in **Figure 4**, we computed the maximum of the features for each subregion to represent the final features. Most of the features were compliant with the Imaging Biomarker Standardization Initiative (IBSI), and all features were extracted using the Irradiomics Tool (version 3.0.1) (23).

To assess the robustness of the image features, we performed a test-replicate analysis and an interrater analysis to ensure that the selected features were not affected by segmentation uncertainty. In the test-replicate analysis, one rater performed two separate segmentations on each set with 30 randomly assigned patients, whereas the interrater evaluation consisted of two raters independently segmenting the VOI subregions of another set of 30 randomly assigned patients. Features extracted from multiple segmentation subregions were evaluated using the ICC, and features with an ICC ≥ 0.85 were considered robust to segmentation uncertainty. After initial screening using the ICC, all the features were standardized to ensure that they were normally distributed. Then, p-values were calculated for all the imaged features using a t-test. Only radiomic features with a p-value < 0.05 were retained. Features of high reproducibility were then analyzed for strong correlations using Pearson correlation. If any two features correlated more than 0.9, only one feature was left standing. A recursive deletion strategy was used, removing the most redundant features at each step to maximize feature representativeness while reducing redundancy. The LASSO regression model was used to select the final set of features to construct the radiomic signature. Based on the regularization weight λ, LASSO regression shrinks the regression coefficients to 0, effectively setting many coefficients for irrelevant features to 0. The ideal λ was determined using 10-fold cross-validation, selecting the value associated with the lowest mean standard error.

Our comparison of the capabilities of various types of tumor region analysis: tumor region as a whole (Intra) and tumor habitat (Habitat) analysis in a PCR prediction task.

### Establishment of the intraradiomics signature (Rad)

Machine learning approaches were used to obtain the final features after Lasso feature screening. We used commonly used machine learning models, such as XGBoost for tree models and logistic regression (LR) for linear models, to build the risk model.

### Establishment of habitat signature(Habitat)

Since the characterization of the internal tumor habitat relies on unsupervised clustering algorithms, we cannot be sure that clusters with the following centers have the same physical meaning. We calculated an average for the features to mitigate this effect. In addition, because of the unsupervised nature of the clustering, the feature selection process for the habitat feature did not include ICC scoring, although the other configurations mirrored those of the intramodel.

### Use of the clinical signature

To investigate the role of clinical features, we used all available clinical parameters for modelling. Owing to the limited number of clinical features, no selection was made during the modelling phase. All other procedures were consistent with the radiomic and habitat modelling workflow.

### Development of a radiomics nomogram

By integrating clinical features and handcrafted habitat-based radiomic signatures, we developed a radiomic nomogram. Receiver operating characteristic (ROC) curves were used to assess the diagnostic performance of the radiomic nomogram in the test cohort. Calibration curves were generated to assess the calibration accuracy, and the Hosmer–Lemeshow goodness-of-fit test was employed to evaluate the calibration ability. Decision curve analysis (DCA) was also performed to evaluate the clinical benefit of the use of the predictive models.

## Results

### Baseline data

We conducted statistical tests to assess the normality of the clinical features, employing the Shapiro−Wilk method. We used the t test for significance analysis for characteristics that followed a normal distribution. For nonnormally distributed characteristics, we used the test to compare clinical features. To identify significant clinical features, we employed both univariate and stepwise multiple regression analyses for feature selection. All analyses were performed using Python v.3.7.12 and statsmodels v. 0.13.2. The machine learning models were developed based on the scikit-learn v1.0.2 interface. **Table 1** displays the baseline features of the patients.

**Table 1:** Baseline features of the patients. The Eastern Cooperative Oncology Group (ECOG) categorizes patients’ activity status into a total of 6 grades from 0 to 5. Surgery is feasible for patients with a general physical status of 0-1.

feature_name	PCR	none-PCR	P-value	PCR	none-PCR	P-value
age	68.78±6.78	65.55±8.21	0.121	62.50±7.23	65.95±7.97	0.598
Tumor_length	5.87±2.38	7.02±2.42	0.037	4.55±1.80	6.33±2.10	0.09
Weight	58.94±9.46	60.82±10.64	0.609	62.33±10.58	60.19±11.18	0.861
Height	163.72±8.09	164.84±7.36	0.771	165.50±7.42	165.95±6.95	1.0
Gender			1.0			0.853
male	14(77.78)	51(76.12)		4(66.67)	17(80.95)	
female	4(22.22)	16(23.88)		2(33.33)	4(19.05)	
ECOG_score			0.645			0.318
0	3(16.67)	17(25.37)		3(50.00)	4(19.05)	
1	15(83.33)	50(74.63)		3(50.00)	17(80.95)	
Tumor_location			0.079			0.643
upper	1(5.56)	4(5.97)		0 (0.0)	0 (0.0)	0(0.0)
mid	13(72.22)	39(58.21)		4(66.67)	18(85.71)	
Distal	2(11.11)	23(34.33)		2(33.33)	3(14.29)	
Gastroesophageal junction	2(11.11)	1(1.49)		0 (0.0)	0 (0.0)	0 (0.0)
T_stage			1.0			1.0
3	18(100.00)	66(98.51)		6(100.00)	20(95.24)	
4	0 (0.0)	1(1.49)		0 (0.0)	1(4.76)	
N_stage			0.325			0.916
1	14(77.78)	40(59.70)		2(33.33)	6(28.57)	
2	3(16.67)	16(23.88)		2(33.33)	9(42.86)	
3	1(5.56)	11(16.42)		2(33.33)	6(28.57)	
Neoadjuvant therapy cycle			0.026			0.778
2	8(44.44)	48(71.64)		5(83.33)	14(66.67)	
3	9(50.00)	19(28.36)		1(16.67)	7(33.33)	
4	1(5.56)	0 (0.0)		0 (0.0)	0 (0.0)	0 (0.0)
Histological_types			0.659			1.0
Squamous cell carcinoma	18(100.00)	64(95.52)		6(100.00)	20(95.24)	
Adenocarcinoma	0 (0.0)	1(1.49)		0 (0.0)	0 (0.0)	0 (0.0)
Other type	0 (0.0)	2(2.99)		0 (0.0)	1(4.76)	

### Use of clinical features

We performed univariate analysis on all the clinical features and calculated the odds ratio (OR) and corresponding p-value for each feature. Among these variables, the number of neoadjuvant therapy cycles had p-values less than 0.05 and was therefore chosen to construct the nomogram. The p-value for the neoadjuvant therapy cycle was 0.014, which was statistically significant (**Supplementary Table S1**, **Supplementary Figure S1**). The correlation matrix presented in **Supplementary Figure S2** depicts the relationships among different clinical features.

### Extraction of habitat features

Altogether, 1834 hand-crafted radiomic features were extracted. They were categorized into shape, first-order and texture categories. Among these, there were 360 first-order features, 14 shape features, and various texture features. The feature extraction process utilized an in-house program implemented in PyRadiomics (http://pyradiomics.readthedocs.io) (**Supplementary Figure S3**).

**Figure 5:**
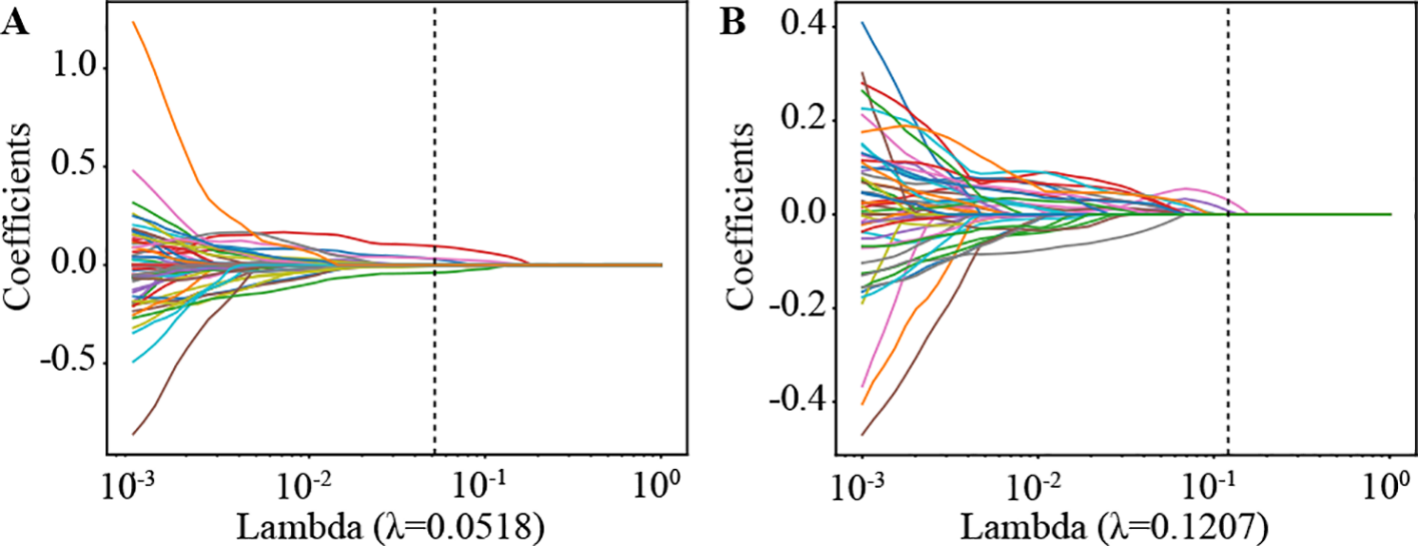
Coefficients of lasso in 10 fold cross validation in Radiomics Signature and Habitat Signature. **(A)** Lasso Coefficients for radiomics signature. **(B)** Lasso Coefficients for habitat signature.

We then established radiomic features through the application of Lasso with 10-fold cross-validation to select tumor features and habitat-related features, as depicted in **Figure 5**.

**Supplementary Figure S4** shows the MSE path of LASSO in a 10-fold cross validation of the Radiomics Signature and Habitat Signature.

For the training set, we applied a 5-fold cross-validation. The cross-validation results clearly revealed that the models based on habitat segmentation significantly outperform traditional radiomic models (**Supplementary Figure S7**).

**Figure 6:**
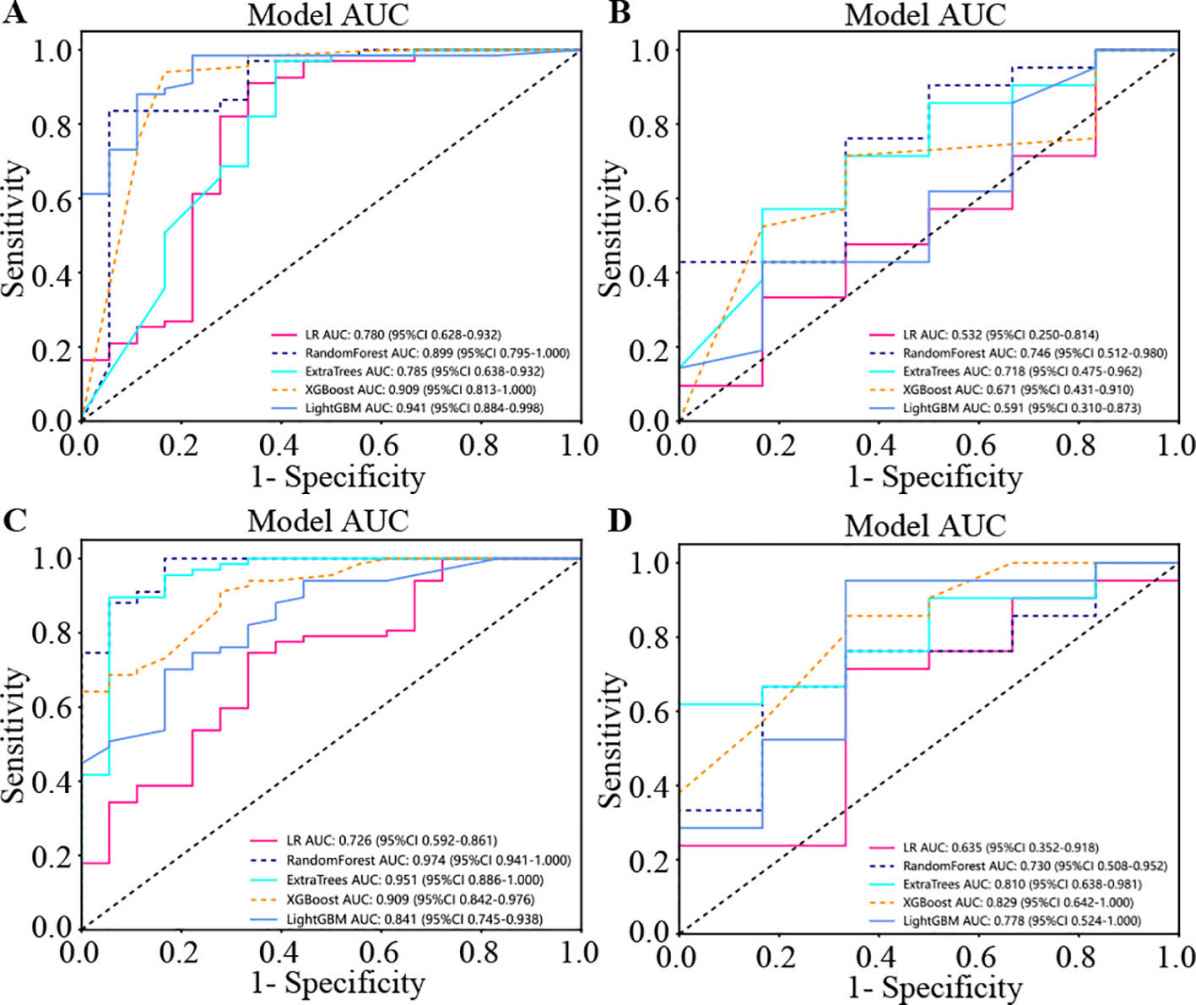
The four presented figures correspond to the performance of the models on intratumoral training and testing datasets, as well as habitat analysis training and testing datasets across various models. The outcomes demonstrate that integrating habitat analysis into the models results in a significant enhancement. **(A)** Indicates the performance of model on the intratumoral training set. **(B)** Indicates the performance of model on the intratumoral test set. **(C)** Indicates the performance of different models on the habitat analysis training set. **(D)** Indicates the performance of different models on the habitat analysis test set.

The four presented figures correspond to the performance of the models on intratumorally training and testing datasets, as well as habitat analysis training and testing datasets across various models. The outcomes demonstrate that integrating habitat analysis into the models results in a significant enhancement (**Figure 6**).

### Signature comparison

#### Model efficiency

The model based on habitat outperforms traditional radiomic models. Additionally, when this model is combined with clinical features, it achieves enhanced predictive accuracy.

**Figure 7:**
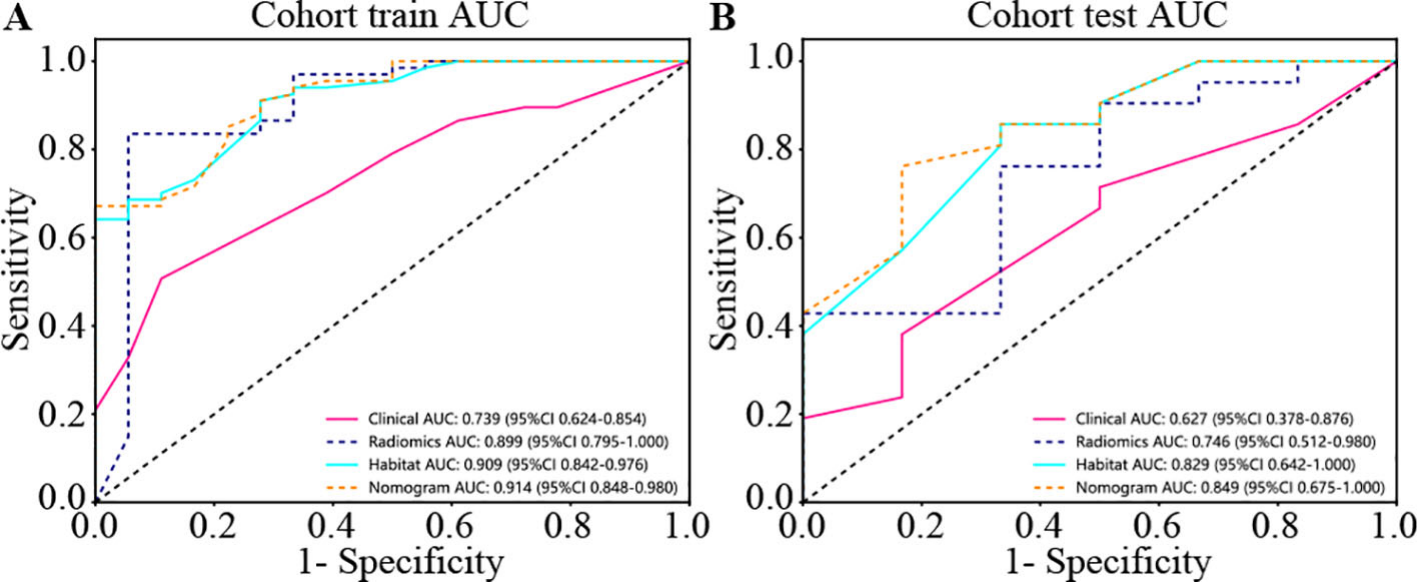
Different signatures’ AUROC on test cohort. **(A)** represents AUC of each model in the training sets. **(B)** represents AUC of each model in the test sets. The model based on habitat outperforms traditional radiomics models. Nomogram models are considered better performers, showing higher accuracy and AUC values across both the training and test sets. As can be seen from the curve, the AUC of Nomogram is the largest.

We produced AUROCs for different features in the test cohort and compared the efficacy of each model. In the test set, the sensitivity of habitat analysis is 66.7%, the specificity is 90.5%, and the sensitivity is the highest among all models. However, the sensitivity and specificity of the traditional radiomics model are only 33.3% and 95.2%, which is the highest specificity among all models. In the nomogram model, both sensitivity and specificity are high. The AUC of nomogram model is 0.914 in training set and 0.849 in test set, which is obviously higher than other models, and it is considered as better performance, showing higher accuracy and AUC value in training and test set. As can be seen from the curve, the AUC of the nomogram is the largest (**Figure 7**, **Table 2**).

**Table 2:** Metrics on different signature. With higher accuracy and AUC values, nomogram models are considered to be better performers.

Signature	Accuracy	AUC	95% CI	Sensitivity	Specificity	PPV	NPV	Cohort
Clinical	0.588	0.739	0.6244 - 0.8540	0.889	0.567	0.327	0.944	Train
Radiomics	0.859	0.899	0.7945 - 1.0000	0.944	0.836	0.607	0.982	Train
Habitat	0.718	0.909	0.8418 - 0.9758	1.000	0.642	0.429	1.000	Train
Nomogram	0.741	0.914	0.8483 - 0.9801	1.000	0.672	0.450	1.000	Train
Clinical	0.630	0.627	0.3779 - 0.8761	0.500	0.667	0.300	0.824	Test
Radiomics	0.815	0.746	0.5120 - 0.9801	0.333	0.952	0.667	0.833	Test
Habitat	0.815	0.829	0.6423 - 1.0000	0.667	0.857	0.571	0.900	Test
Nomogram	0.815	0.849	0.6752 - 1.0000	0.500	0.905	0.600	0.864	Test

#### Calibration curve

The HL test statistics represent the degree of discrepancy between the predicted probabilities and the observed outcomes. A higher HL test statistic indicates better calibration, suggesting that the models’ predicted probabilities align closely with the actual outcomes. In this context, the nomogram model demonstrates the highest calibration performance, with an HL test statistic of 0.321 (**Supplementary Figure S5**, **Supplementary Table S2**).

#### DeLong test

When the training and testing sets are examined, the nomogram, which integrates both clinical and machine learning outcomes, clearly shows superior model performance. In the training cohort, all the signatures demonstrated significant enhancement compared with the clinical signature (p-value < 0.05). However, the improvement in the test set is not as pronounced. This could be attributed to the limited impact of the clinical model in the linear fusion process (**Supplementary Figure S6**).

### Clinical use

**Figure 8:**
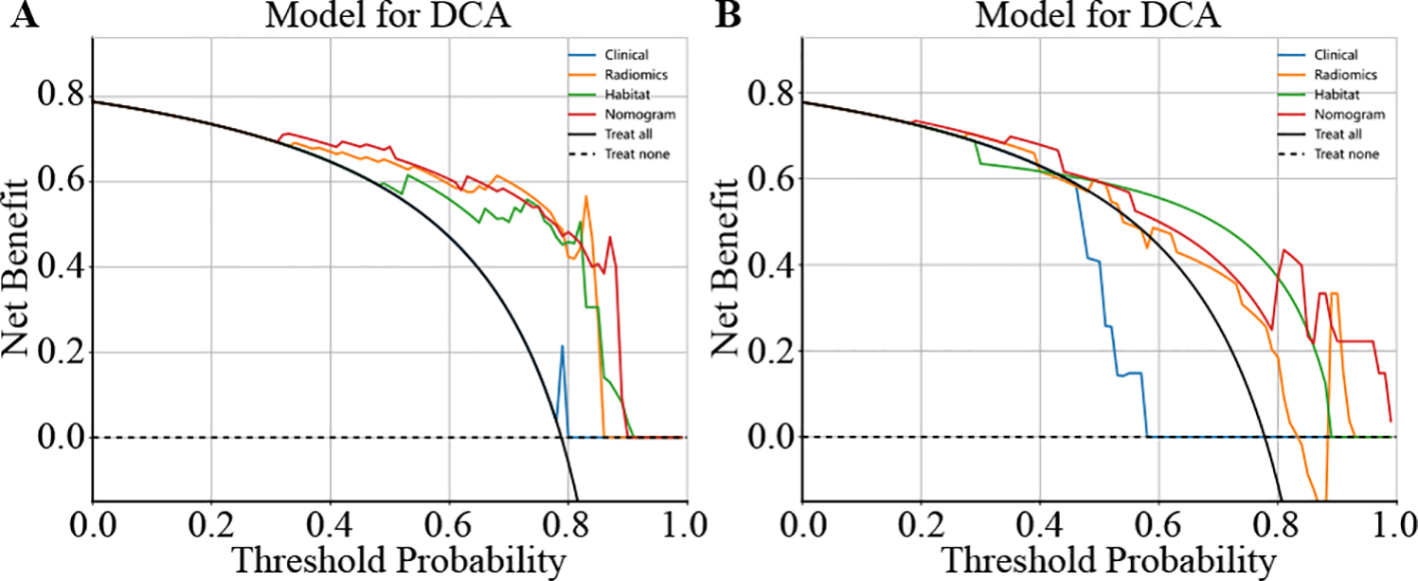
Different signatures’ decision curve on test cohort. **(A)** shows the DCA for the training set. **(B)** shows the DCA for the test set.

We used decision curve analysis (DCA) to assess the effectiveness of the model (**Figure 8**). The results indicate that our fusion model (nomogram) yields noticeable benefits on the basis of the predicted probabilities. Additionally, compared with other signatures, it has greater potential for obtaining net benefit.

### Establishment of the nomogram

**Figure 9:**
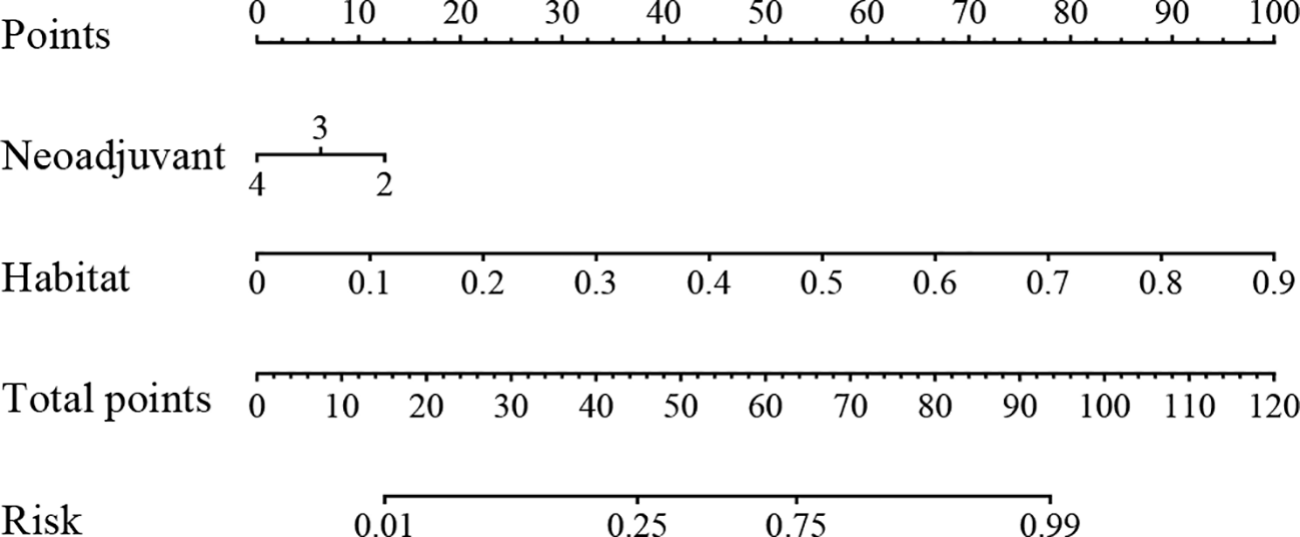
The nomogram for clinical use.

We integrated all the clinical features with the signature to construct a nomogram, which facilitates clinical utilization (**Figure 9**).

## Discussion

In this study, we developed a model that combines habitat analysis, radiomics scores and clinical variables and successfully predicts the prognosis of patients receiving neoadjuvant therapy. Our findings can be used to assess the benefits of NACI prior to treatment and provide patients who are insensitive to NACI with the opportunity to adjust to an appropriate strategy in a timely manner.

In our study, the probability of PCR occurring after patients were treated with NACI was 21%. A limited surgical strategy can be used to minimize surgical complications and economic burdens in patients who are expected to achieve PCR after NACI. To assess the response to NACI, a widely used clinical tool to evaluate changes in tumor size is RECIST (24, 25). Several studies have shown that the esophageal cancer response to NACI is associated with clinical TNM stage and Ki-67 (8, 26). However, the efficacy of NACI is not accurately predicted by RECIST or traditional clinicopathological features. Radiomics has been shown to be an excellent tool for assessing the response of EC patients to NACI in several studies (27–30). Hou et al. used δ18F-fluorodeoxyglucose PET-CT to predict radiation pneumonitis in patients with esophageal squamous cell carcinoma receiving neoadjuvant chemotherapy (28). Wang et al. reported that CT-based radiomics has predictive value for PCR after NACI in patients with esophageal squamous cell carcinoma (30). Intratumoural heterogeneity can be reflected to some extent by the unique texture and spatial greyscale patterns of radiomic features extracted from CT images. However, these theoretical assumptions have not been fully elucidated by habitat analysis studies. Our study has several significant advantages over previous studies: we systematically studied the tumor microenvironment. By integrating information from these subregions, we achieved good results in the prediction task of PCR. In addition, we compared image-based habitat analysis under different algorithmic models in detail.

Habitat analysis methods are now used in many studies of medical images. Wang et al. applied PET/CT habitat radiomics analysis to predict progression-free survival (PFS) status and Ki-67 (31). WEI et al. used MRI habitat radiomic features to predict the preoperative MGMT methylation status and validated its value for assessing the outcome of chemotherapy (32). Habitat-based radiomic analysis for evaluating immediate response in colorectal cancer lung metastases treated by radiofrequency ablation, Huang et al. (33). However, to our knowledge, no studies have combined radiomics with habitat analysis to predict the efficacy of neoadjuvant therapy for esophageal cancer.

**Figure 10:**
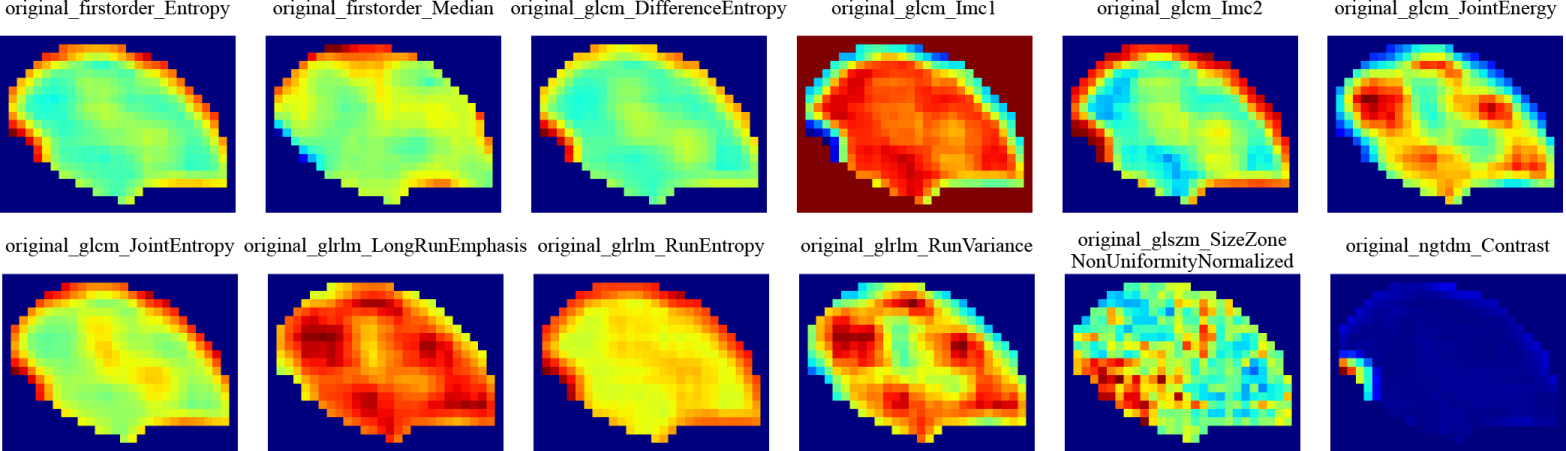
Intratumoral subregions were clustered and regions with the same characteristics were color-coded.

Local features are computed for each of the voxels inside the VOI, including metrics such as local entropy and energy values, to form a feature vector that encompasses the attributes of different voxels. We predetermined three clustering centers as the number of habitat areas. We believe that experimenting with different numbers of clustering centers may enhance the efficiency of the model (**Figure 10**). This finding suggests that the tumor habitat in CT images typically reflects the activity profile of active tumor cells and is strongly associated with tumor progression and death (31, 34, 35). While some of this was due to a potential partial volume effect, it is worth noting that some of the low-activity CT regions were usually located at the tumor margin, suggesting that the heterogeneity of the tumor margin on CT images, which may be caused by inflammation, is also important for prognostic assessment. The higher the entropy value of the lung cancer margins is, the worse the prognosis, as previous studies have shown (36).

This study has several limitations. First, this was a retrospective study with potential limitations associated with retrospective data collection, including selection bias and sample representativeness. To investigate the effect of neoadjuvant therapy for esophageal cancer based on CT radiomics prediction, further prospective analyses should be conducted. Second, manual tumor segmentation by different experts may affect the stability of radiomic features. Although we addressed this issue by selecting features with intraclass correlations above 0.85, it is essential to adopt an automated and accurate tumor segmentation method that not only improves the efficiency of quantitative image analysis but also ensures the stability and consistency of the analysis process.

In summary, this model combines clinical, radiomic and habitat radiomic features and has the best ability to predict PCR in esophageal cancer patients. Future studies should incorporate multimodal data and increase external validation.

Habitat analysis can quantitatively analyze the correlation between habitat area and clinical data by extracting relevant features from medical images, which provides a new method for noninvasive evaluation of tumor types, prediction of metastasis risk, identification of gene status and prediction of prognosis. However, there are still several challenges in this field, such as small sample sizes, a lack of external verification and time-consuming segmentation and extraction. Therefore, further exploration is needed in the field of tumor research. With the gradual standardization of medical image data collection, processing, feature extraction and analysis, the uncertainty of research results will also be reduced. Moreover, with the continuous development of artificial intelligence and imaging, future habitat analysis can extract more accurate habitat features from automatically segmented regions of interest to display tumors and peritumoral conditions more comprehensively.

## Conclusions

We established and evaluated a model of habitat radiomic analysis in patients with EC and applied this model to predict the efficacy of NACI in EC patients. The developed model has excellent predictive power and may improve individualized treatment for EC patients. We packaged the developed algorithm into a Web system, which can direct users to directly input the patient’s image data and judge the patient through our trained model. In clinical practice, doctors can combine the judgement of the AI system and make comprehensive diagnostic conclusions as much as possible. We also intend to use habitat analysis to create a new method for noninvasive evaluation of tumor types, prediction of metastasis risk, identification of gene status and prediction of prognosis.

## Data Availability

The original contributions presented in the study are included in the article/**Supplementary Material**. Further inquiries can be directed to the corresponding author/s.

## Supplementary material

Supplementary Figure 1
**(A)** OR of clinical features in univariable analysis. **(B)** OR of clinical features in multivariable analysis.

Supplementary Figure 2 depicts the relationships among different clinical features.

Supplementary Figure 3 Visually presents the proportion of each group of handcrafted features.

Supplementary Figure 4 MSE path of lasso in 10 fold cross validation in Radiomics Signature and Habitat Signature.

Supplementary Figure 5 Different signatures’ calibration curve on test cohort.

Supplementary Figure 6 Delong et al. of different signature.

Supplementary Figure 7 5 fold cross validation of Radiomics and Habitat, it leads to improvements of nearly 0.1 AUC in almost all models.
