# Supplementary figures and images for: CT-based habitat radiomics for predicting treatment response to neoadjuvant chemoimmunotherapy in esophageal cancer patients
Source: Front Oncol. 2024 Dec 3;14:1418252. doi: 10.3389/fonc.2024.1418252 (PMC11649542; doi:10.3389/fonc.2024.1418252)

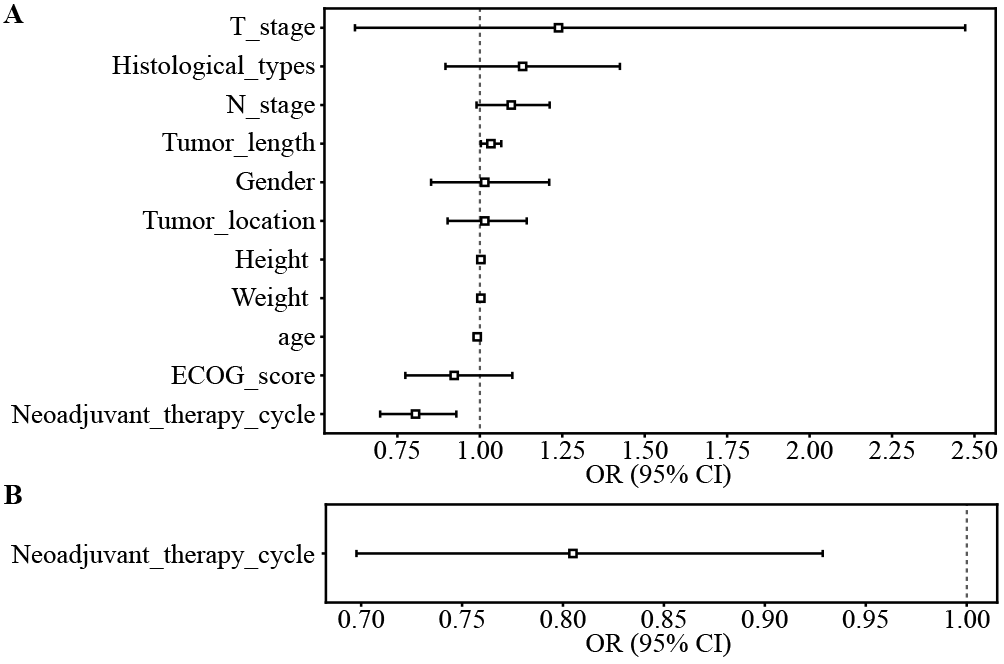

Supplement: Supplementary Figure 1 — (A) OR of clinical features in univariable analysis. (B) OR of clinical features in multivariable analysis. [file Image1.tif]

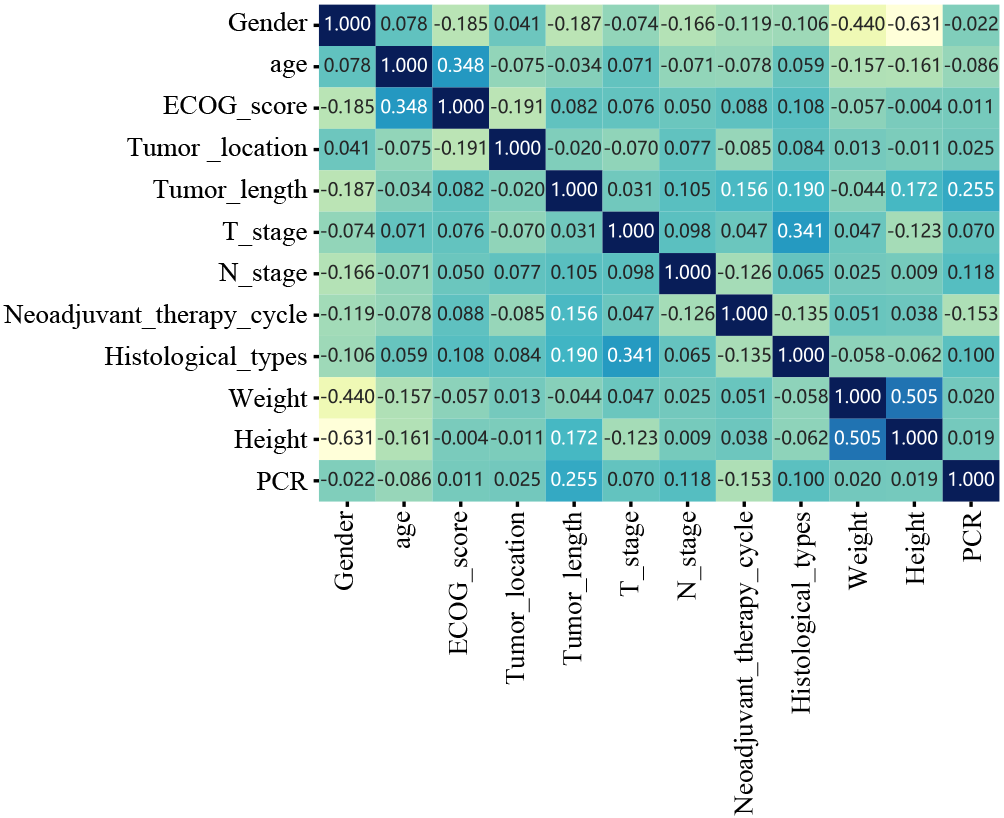

Supplement: Supplementary Figure 2 — depicts the relationships among different clinical features. [file Image2.tif]

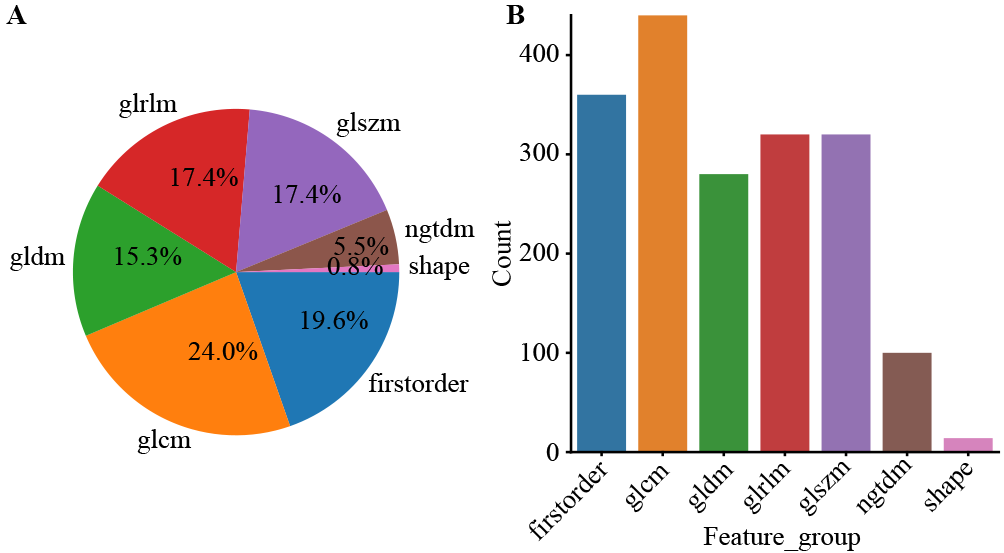

Supplement: Supplementary Figure 3 — Visually presents the proportion of each group of handcrafted features. [file Image3.tif]

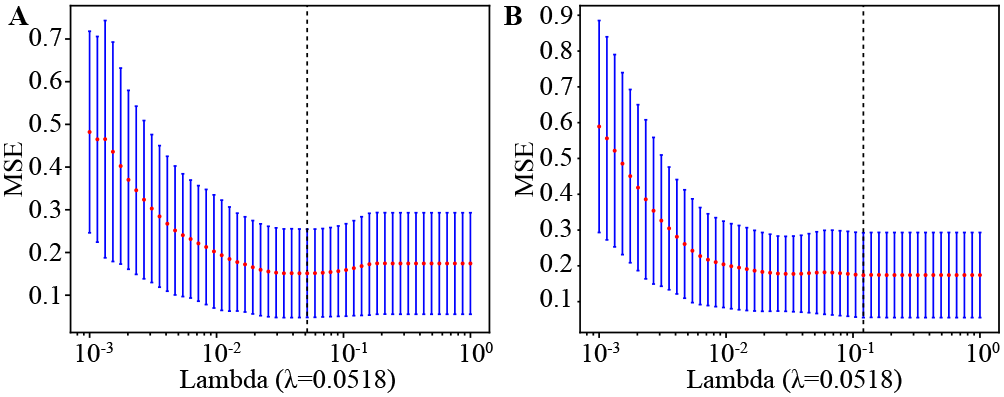

Supplement: Supplementary Figure 4 — MSE path of lasso in 10 fold cross validation in Radiomics Signature and Habitat Signature. [file Image4.tif]

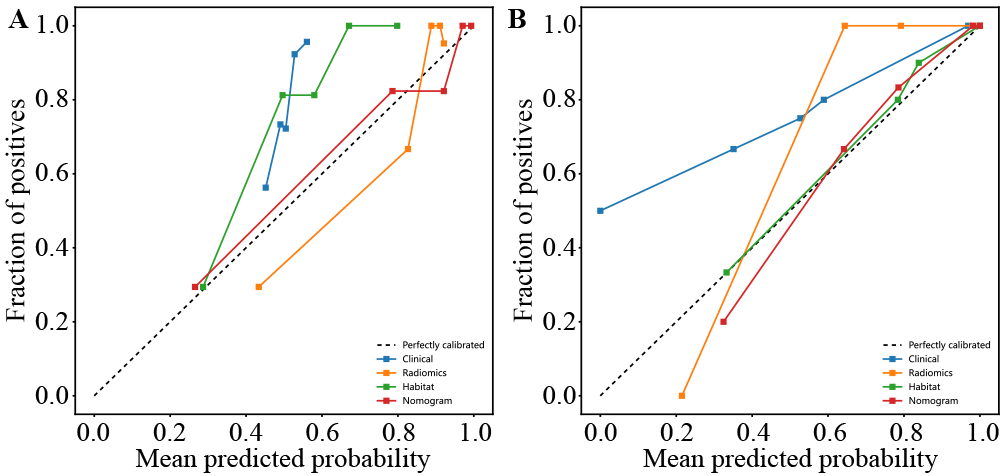

Supplement: Supplementary Figure 5 — Different signatures’ calibration curve on test cohort. [file Image5.tif]

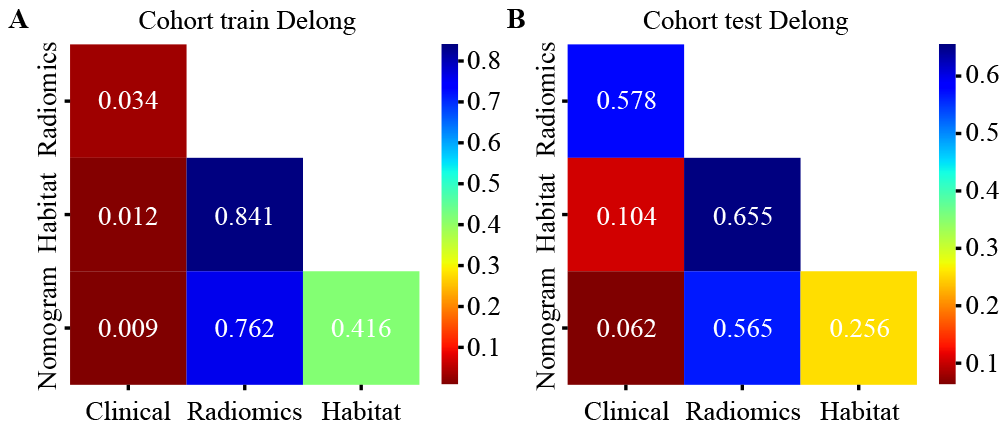

Supplement: Supplementary Figure 6 — Delong et al. of different signature. [file Image6.tif]

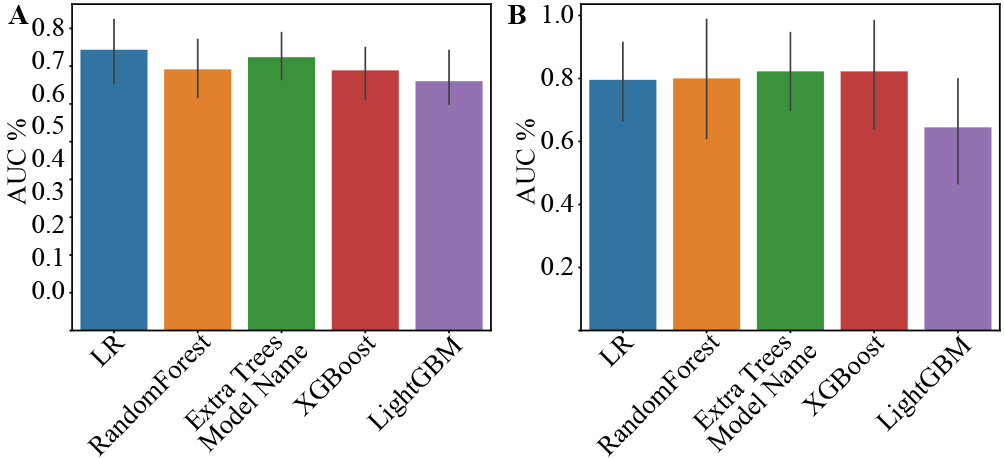

Supplement: Supplementary Figure 7 — 5 fold cross validation of Radiomics and Habitat, it leads to improvements of nearly 0.1 AUC in almost all models. [file Image7.tif]
