# Supplementary material for: CT-based habitat radiomics for predicting treatment response to neoadjuvant chemoimmunotherapy in esophageal cancer patients
Source: Front Oncol. 2024 Dec 3;14:1418252. doi: 10.3389/fonc.2024.1418252 (PMC11649542; doi:10.3389/fonc.2024.1418252)
Supplement: Supplementary file 8 [file Table1.docx]

| **feature_name** | **OR** | **OR lower 95%CI** | **OR upper 95%CI** | **p-value** | **OR** | **OR lower 95%CI** | **OR upper 95%CI** | **p-value** |
| --- | --- | --- | --- | --- | --- | --- | --- | --- |
| Neoadjuvant therapy cycle | 0.805 | 0.698 | 0.929 | 0.014 | 0.805 | 0.698 | 0.929 | 0.014 |
| ECOG_score | 0.922 | 0.774 | 1.099 | 0.445 |  |  |  |  |
| age | 0.992 | 0.982 | 1.001 | 0.130 |  |  |  |  |
| Weight | 1.003 | 0.996 | 1.010 | 0.499 |  |  |  |  |
| Height | 1.003 | 0.993 | 1.013 | 0.578 |  |  |  |  |
| Tumor_location | 1.015 | 0.902 | 1.142 | 0.834 |  |  |  |  |
| Gender | 1.016 | 0.852 | 1.210 | 0.885 |  |  |  |  |
| Tumor_length | 1.033 | 1.003 | 1.065 | 0.074 |  |  |  |  |
| N_stage | 1.095 | 0.990 | 1.212 | 0.138 |  |  |  |  |
| Histological_types | 1.129 | 0.896 | 1.425 | 0.386 |  |  |  |  |
| T_stage | 1.239 | 0.621 | 2.472 | 0.607 |  |  |  |  |

## **Supplementary Table S1** Univariable and Multivariable Analysis of clinical features.The p-value for the neoadjuvant therapy cycle is 0.014, which is statistically significant.
