# Supplementary material for: CT-based habitat radiomics for predicting treatment response to neoadjuvant chemoimmunotherapy in esophageal cancer patients
Source: Front Oncol. 2024 Dec 3;14:1418252. doi: 10.3389/fonc.2024.1418252 (PMC11649542; doi:10.3389/fonc.2024.1418252)
Supplement: Supplementary file 9 [file Table2.docx]

**Supplementary Table S2.** Hosmer-Lemeshow (HL) test statics for each signature.

| **Clinical** | **Radiomics** | **Habitat** | **Nomogram** |
| --- | --- | --- | --- |
| 0.756 | 0.052 | 0.368 | 0.267 |
| 0.653 | 0.112 | 0.219 | 0.321 |
